# Recent Advances in Natural Gum-Based Biomaterials for Tissue Engineering and Regenerative Medicine: A Review

**DOI:** 10.3390/polym12010176

**Published:** 2020-01-09

**Authors:** Reza Mohammadinejad, Anuj Kumar, Marziyeh Ranjbar-Mohammadi, Milad Ashrafizadeh, Sung Soo Han, Gilson Khang, Ziba Roveimiab

**Affiliations:** 1 Neuroscience Research Center, Institute of Neuropharmacology, Kerman University of Medical Sciences, Kerman 7619813159, Iran; r.mohammadinejad87@gmail.com; 2 School of Chemical Engineering, Yeungnam University, 280 Daehak-Ro, Gyeongsan 38541, Korea; 3 Department of Textile, Engineering Faculty, University of Bonab, Bonab 5551761167, Iran; ranjbar.aut@gmail.com; 4 Department of Basic Science, Faculty of Veterinary Medicine, University of Tabriz, Tabriz 5166616471, Iran; dvm.milad73@yahoo.com; 5 Department of Polymer Nano Science and Technology, Department of BIN Fusion Technology and BK-21 Polymer BIN Fusion Research Team, Chonbuk National University, Dukjin, Jeonju 54896, Korea; gskhang@jbnu.ac.kr; 6 Department of Biological Sciences, and Department of Physics and Astronomy, University of Manitoba, Winnipeg, MB R3T 2N2, Canada; zibarovei@gmail.com

**Keywords:** natural gums, nanofibrous scaffolds, hydrogels, nanocomposites, extracellular matrix, tissue engineering

## Abstract

The engineering of tissues under a three-dimensional (3D) microenvironment is a great challenge and needs a suitable supporting biomaterial-based scaffold that may facilitate cell attachment, spreading, proliferation, migration, and differentiation for proper tissue regeneration or organ reconstruction. Polysaccharides as natural polymers promise great potential in the preparation of a three-dimensional artificial extracellular matrix (ECM) (i.e., hydrogel) via various processing methods and conditions. Natural polymers, especially gums, based upon hydrogel systems, provide similarities largely with the native ECM and excellent biological response. Here, we review the origin and physico-chemical characteristics of potentially used natural gums. In addition, various forms of scaffolds (e.g., nanofibrous, 3D printed-constructs) based on gums and their efficacy in 3D cell culture and various tissue regenerations such as bone, osteoarthritis and cartilage, skin/wound, retinal, neural, and other tissues are discussed. Finally, the advantages and limitations of natural gums are precisely described for future perspectives in tissue engineering and regenerative medicine in the concluding remarks.

## 1. Introduction

Bio-based materials as matrices in tissue engineering propose significant choices in regulating the frame, microstructure (morphology), and biointerfaces (chemistry) as reasonable substitutes that mimic native extracellular matrix (ECM) systems. Furthermore, these characters allow better regulation of the compound roles including the mechanical possessions in the gel, fiber, and porosity of scaffolds structure. The biodegradability of biopolymers is largely significant to assist in regulating the level and limit of the cell and tissue remodeling in vitro or in vivo. The potency to genetically redevize these natural polymers to bioengineer suitable characters in regulating the cell responses and interaction is another remarkable characteristic that proposes the substantial realization of both the fundamental insight into chemistry–structure–function relationships and the upstanding advantages. When implanted in vivo, natural polymers exhibit structural compatibility much similar to the biological molecules in organisms, thereby decreasing the possibility of an immune response. Therefore, some polysaccharides are non-immunogenic or they show low immunogenicity as compared to synthetic polymers. Biopolymer-based scaffolds for tissue engineering can directly affect the functional aspects of the formed tissues and will play a progressively and continuously significant role in this field [1,2].

**Figure 1:**
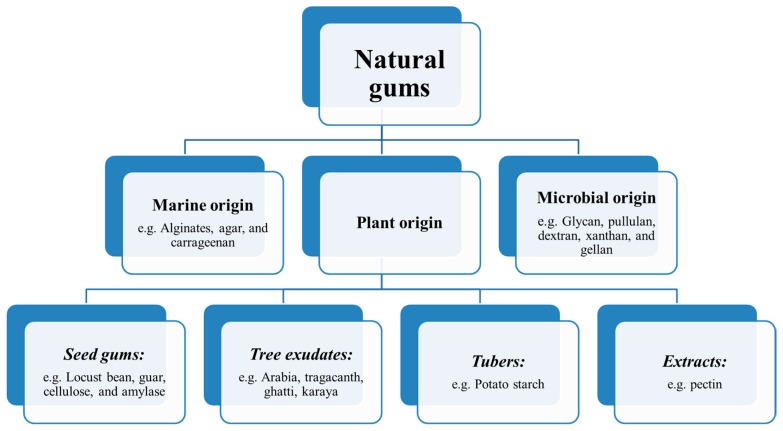
Origin of the natural gums.

Polysaccharides are an example of the highly effective biomaterials used in the tissue engineering, which are formed from a large number of monomeric units with glycosidic bonds and are characterized by their prominent features, from cell signaling to the detection of immunity. Polysaccharides are obtained as the most abundant organic polymers from different natural sources (see Figure 1) of plants, animals, microbials, and algae, and their molecules consist of a ring-shaped conjugation of monosaccharides through etheric bonds [3]. Based on their structures and properties, polysaccharides have been used in various applications, such as textile, paper, and the adhesive industry, and over the past two decades, extended their use in new areas such as the construction of specific membranes, coatings, drug release systems, emulsifiers, and cellular scaffolds [4].

As described above, polysaccharides can be found in the body of many living organisms [5]. Natural gums as polysaccharides (i.e., composed of sugars) are capable of causing an increment in a solution viscosity even at smaller concentrations. They are low-cost, chemically inert, biocompatible, nontoxic, odorless, and most abundant. As these gums are water-soluble, they are also known as hydrocolloids. First, they serve as the sources of reserve nutrients, structural entities, and water-binding components. Second, they serve as ingredients in the food by controlling the impact of texture, shape, water-binding, and sensory effects. Finally, polysaccharides act as nutrients and dietary fiber sources [6]. Due to these properties, gums have been used in various industrial and biomedical applications [7,8]. They have been applied as stabilizers and gelling, thickening, and emulsifying agents due to their water-binding capacity, rheological behavior, and encapsulation of different materials like flavors, aromas, and nutraceutical agents, including the ability to produce films or gels. Most often, plant-based gums are found inside the seed-coatings or in the woody elements of the plants. Sometimes, they are found in the plant cell walls, tree exudates, and tuber/roots. Several plants produce these gums in response to a mechanism of protection against mechanical injury or microbial injury [9].

## 2. Natural Gums as Renewable Bio-Based Materials

Polysaccharides are among the most dominant renewable resources for human beings. Bio-based materials are commonly proposed to be more sustainable than typical petrochemical materials because they are created from renewable instead of non-renewable raw materials [10]. There are a large number of hydrophilic moieties having hydroxyl and other polar functional groups in the chemical structure of natural gums forming hydrogen bonds that play major roles in their film formation process. The mechanical, thermal, rheological, optical and film-forming characteristics are influenced by their structural functionality.

Gums possess particularly three important properties: (a) prominent capability to attract and bind water to form a viscous solution; (b) limited contribution of calories, notably in the large intestine, due to very limited absorption and digestion in the body (act as a fiber); and (c) film-forming ability. The film-forming characteristics of natural gums depend on intermolecular forces such as crosslinking, electrostatic, hydrophobic, ionic interactions, or inter-/intra-molecular hydrogen bonding. In some cases, natural polymers have been chemically modified through the crosslinking of functional groups and the hydrolysis of polysaccharides [11,12].

Based on their surface charges, the gums are categorized into anionic (e.g., tragacanth, arabic, karaya, gellan, agar, pectin, xanthan, alginate, and carrageenan gum), cationic (e.g., modified guar gum), and non-ionic (e.g., arabinans, amylase, locust bean gum, tamarind gum, cellulose), respectively. Further, gums have also been classified according to their structure as linear chains (e.g., pectin, amylase, cellulose, and locus bean gum,) and branched chains (namely short branching as guar gum and long branching as amylopectin, gum tragacanth, karaya gum, gum arabic) [9,13,14].

## 3. Origin of Natural Gums

Natural gums can be categorized according to their sources and chemical structures. (a) Marine (sea weeds) origin: agar, alginate, carrageenan; (b) microbial origin (fungi, bacteria, algae): pullulan, glycan, dextran, gellan gum, dextran; and (c) plant origin: (i) seed gums (e.g., guar gum, locust bean gum, starch, amylase, cellulose); (ii) tree exudates: gum arabia, gum tragacanth, ghatti gum, karaya gum; (iii) tubers-Potato starch (e.g., Konjac); and (iv) extracts-pectin [15]. Gums are largely obtained from the following three groups: (a) the endosperm of some seeds (e.g., guar gum, locust bean gum); (b) plant exudate gums (e.g., gum tragacanth); and (c) tree or shrub exudates (e.g., gum arabic, karaya gum). Among known natural gums, only a few important gums have been applied in the food industry [16,17,18].

Seed gums are widely distributed in nature as a galactomannan and come under the class of hydrocolloids to strengthen matrix structures. They act as a reserve polysaccharide extracted from the seeds. The structure of galactomannan differs from the mannose/galactose ratio and the proportion of galactose residues in the main chain. It has been found that the variation in the mannose/galactose ratio causes significant changes in galactomannan property and its interactions with other polysaccharides. Plant exudate gums are obtained from the bark of trunk, branch, and fruit of trees in response to the environmental stress or injury. Exudates gums are the oldest types and have usually been used as thickener, stabilizer, rheology modifier, dietary fiber, and fat replacers [19].

## 4. Physico-Chemical Properties of Natural Gums

The extensive use of natural polymers in different fields has led to the development of products with new technologies in the manufacturing of various material systems through changes in the structure or biochemical and mechanical properties of these materials. The great chemical and biological characteristics of natural polymers have been highly effective in many fields such as nutrition, medicine, pharmacology, agriculture, and environmental and chemical engineering [20,21]. Several gums are biocompatible and many scientists have continually tried to use them in the preparation of medical systems or medical devices such as wound healing dressings, drug delivery, and artificial tissue engineering scaffolding systems that are very similar to native body tissues (e.g., having a complex microstructure) [22]. Such natural polymers have been considered due to their excellent biocompatibility, biodegradability, nontoxicity, inexpensiveness, and ease of accessibility as compared to synthetic polymers [23]. Different types of natural polysaccharides, such as dextran, gum tragacanth, pectin, guar gum, xanthan gum, carrageenan, etc., have been used for pharmaceutical applications in the form of films, nanofibers, and nanoparticles [24,25,26,27].

Gum tragacanth (GT), an anionic and acidic polysaccharide with high molecular weight (8.4 × 10^5^ Da), complex macrostructure, and durability, has been extensively used in various areas due to its characteristics including biodegradability, nontoxicity, availability in nature, and stability against microbial, heat, and acid attacks. GT is obtained from 15 different species of *Astragalus* and spontaneously secreted from cracks created on the bushes’ body [28]. This branched and heterogeneous biopolymer is non-allergenic, non-carcinogenic, and has no toxic effects [29]. In pharmacy, GT is also used as a gelling agent, suspending agent, and binder in the preparation of pills and medications. Its capability in the prevention of cancer cell growth, wound healing, and other medical applications has been confirmed [30,31,32]. GT is often grown in Central Asia and Eastern countries, including Iran and Turkey, and the best kind of GT is obtained at altitudes of 1300 m and above [33,34]. GT is extracted from the stems and branches of Asiatic species of Astragalus such as *A. adscendens*, *A. gummifer*, *A. brachycalyx*, and *A. tragacantha*. Two components form the composition of the GT, namely tragacanthin and tragacanthic acid or bassorin. The latter portion is insoluble in water but has gelling capability due to swelling. GT is a typical branched arabinogalactan (tragacanthin), a water-soluble portion that produces a colloidal solution in water [35], containing (1→6)- and (1→3)-linked galactose and terminal (1→2)-, (1→3)-, and (1→5)-linked Larabinose. Tragacanthic acid, a pectin type of composition, is the major constituent of GT with structural linkages such as (1→4)-linked α-D-galacturonan substituted at O-3 by β-D-xylose [36,37,38,39]. Because of its desirable properties such as hydrophobicity, ionic nature, and the presence of pH-sensitive and biodegradable groups, it has potential for use in hydrogel release control systems. Consequently, GT can be evaluated for the production of scaffolds used in tissue engineering as a promising material because of its biocompatibility, presence of functional groups and structure similar to that of the body’s polymers, and the presence of specific monosaccharides in its structure.

Xanthan gum (XG), an anionic polysaccharide, produces a viscous solution (derived from its high molecular weight) at low shear rates, which is synthesized by *Xanthomonas* bacteria (a Gram-negative bacteria) [40]. It is stable in a broad spectrum of pH values. Its major chain is similar to that of cellulose (D-glucose units linked together via β(1→4) bonds) and its side chains are composed of alternating residues of D-mannose and D-glucuronic acid in a ratio of 2:1 [41,42]. XG, with extensive purification, is biodegradable and biocompatible, therefore this gum has been applied in various biomedical applications, including the controlled release of drugs, alone or combined with other natural and/or synthetic polymers [43], and tissue regeneration [44].

Dextran is hydrophilic, nontoxic, and biocompatible homopolysaccharide, which is derived bacterially from sucrose with dextransucrase or maltodextrins with dextrinase [45]. This biopolymer mostly contains the α(1→6)-linked d-glucopyranosyl backbone modified with small side chains of D-glucose branches with α(1→2), α(1→3), and α(1→4)-linkage [45]. This polymer with biocompatibility and biodegradability has been extensively used in biomedical and pharmaceutical applications to decrease inflammatory response and stimulate wound healing and perfect skin rehabilitation [45]. Alginate is an anionic biopolymer containing mannuronic acid and glucuronic acid units with a random organization [46]. Glycosidic attachments provide the binding of the units of mannuronic acid and glucuronic acid [47,48,49]. It seems that α and β bonds (1→4) are responsible for the mannuronic acid and glucuronic acid attachment [50]. The special conformations of glucuronic acid determine the rigidity of molecular chains [51,52]. These promising properties of alginate, particularly biocompatibility, have led to its extensive application in nanomedicine and tissue engineering. Further, chitin is a natural polymer extensively found in the shells of insects and fungi, whereas chitosan (CS) is a linear polysaccharide isolated from chitin [53]. The negligible deacetylation of chitin by chemical hydrolysis (alkaline conditions) or enzymatic hydrolysis (chitin deacetylase) results in the formation of CS [54,55]. Similar to alginate, CS has demonstrated great potential in being used in nanomedicine and tissue engineering.

Hyaluronic acid (HA) is an anionic copolymer consisting of units of β-1,4-D-glucuronic acid-β-1,3-N-acetyl-D-glucosamine [56]. HA is widely found in connective, epithelial, and neural tissues [57]. Notably, hydroxyl and carboxylic acid groups have made possible the combination of HA with other compounds [58]. HA has a variety of excellent properties such as biocompatibility and biodegradability [59]. This polymer is applied in medicine for the treatment of a number of pathological conditions such as arthritis. Besides, HA is of interest in drug delivery and tissue engineering [56,60].

Carrageenans (CCG) are a family of linear sulfated polysaccharides that are isolated from red edible seaweeds. The structure of this gum is a linear (1→4)-linked β-D-glucose backbone (as in cellulose) with a trisaccharide side chain on every other glucose at C-3, containing a glucuronic acid residue-linked (1→4) to a terminal mannose unit and (1→2) to a second mannose that connects to the backbone [61]. It can be used in various industrial products due to its non-toxic nature, low-cost, and biocompatible nature [62]. CCG is currently an excellent choice in tissue engineering and regenerative medicine and simulates native glycosaminoglycans because of its preferable gelling reaction mechanism, large number of –OH and –OSO_3_^−^ functional groups, high water-absorption capacity, and other desirable physico-chemical characteristics [63]. Guar Gum (GuG) is a water-soluble and non-ionic galactomannan high molecular weight plant polysaccharide that is extracted from *Cyampsis tetragonolobus* seeds [64] and is composed of linear chains of β-(1-4)-D-mannan, having side chains of α-(1-6) linked galactose [65]. It has been extensively utilized in tissue engineering primarily due to its biodegradability, high biocompatibility, and rheological characteristics [66]. GuG has been used for releasing methotrexate (an anticancer drug) at the targeted site of colon tumor [67]. The advantages of using selenium-GuG in biomedical fields have been reported due to the lack of apoptosis by selenium-GuG and damage of DNA by hydroxyl radical [68]. Selected natural gums, including the above-described gums and their properties, are given in Table 1.

## 5. Various Forms of Scaffolds Based on Gums

For many years, researchers have tried to produce scaffolds with a fibrous network by generating a nanoscale/microscale structure. For this, different fabrication techniques have been developed such as the spinneret-based tunable engineered parameter (STEP) method [94], drawing method [95], self-assembly [96], phase separation freeze-drying, template synthesis, and interfacial polymerization of nanofibers, electrospinning, and other production methods for polymeric scaffolds [97], etc. Among them, the electrospinning technique is the process of producing nanofibrous scaffolds under electrostatic forces and more effective in the preparation of gum-based nanofibers. This is the easiest, most affordable, cheapest, and most commonly used method for fabricating medical nanofibers [98]. The advantages of electrospinning, unlike other scaffolding manufacturing methods, include the production of continuous fibers using a large number of polymers, the change in the morphology and size of the fibers by changing the solvent, and process parameters [99]. However, the electrospinning of natural polymers is not possible because of their high molecular weight (generally in the range of 10^6^ Da), high polydispersity, complex molecular structures, and their polycations or polyanion nature. Therefore, blending them with other synthetic polymers has been performed to help in their ease of spinnability without breaking up into droplets [100]. Hydrogel is a crosslinked hydrophilic polymeric network to produce an elastic structure. Thus, various techniques have been used to construct a crosslinked hydrogel network. Among them, copolymerization/crosslinking free-radical polymerizations are commonly applied to fabricate hydrogels by reacting hydrophilic monomers with multifunctional crosslinkers. In general, hydrogels can be produced from either synthetic, natural, or both types of polymers. The synthetic polymers are hydrophobic, chemically stronger, and durable with a slow rate of degradation compared to natural polymers [101,102,103,104,105]. Hydrogels based on natural polymers have suitable functional groups or can be modified with free-radically polymerizable functional groups [106]. For example, the formulation of methacrylated gellan gum (GeGMA) has been suggested as a second-generation hydrogel for the repairing of cartilage tissues [107]. 

Three-dimensional (3D) printing technologies have gained much attention to fabricate patient-specific scaffolds in tissue engineering and medical fields due to their precise control and ability to print complex architectures based on the anatomy of tissue or organ. Using this technology, various studies have been carried out by using natural polymers and hydroxyapatite (HAp) (e.g., collagen or chitosan and dextran sulfate, or silk fibroin (SF) with HAp) or in their combinations with synthetic polymers (e.g., starch–polycaprolactone/HAp) for tissue engineering applications (e.g., hard tissue regeneration) [108]. Apart from them, alginates as natural gum can be effectively used to produce porous three-dimensional networks for tissue engineering applications. Alginate is highly suitable for 3D printing technologies; especially extrusion-based 3D printing due to its ease of crosslinking ability and that it has widely been used for 3D printing/bioprinting in tissue engineering applications. Alginate in the form of hydrogel has exhibited good printability and excellent biocompatibility for application in vascular, cartilage, and bone tissue printing [109]. Further, gelatin, as a hydrolyzed form of collagen, exhibits excellent biocompatibility and inherent bioactivity that are essential to prepare hydrogels for tissue engineering (e.g., osteochondral regeneration, bone defect) [110,111]. The combination of alginate and gelatin facilitates the printability of polymer inks for better properties. Concentrated alginate/gelatin scaffolds with homogeneous nano-apatite coating were fabricated by 3D printing followed by in situ mineralization for application in bone tissue engineering [112]. In addition, alginate/gelatin constructs reinforced with TiO_2_ and β-tricalcium phosphate fabricated by micro-extrusion-based printing showed enhanced mechanical properties up to 20 MPa of elastic modulus [113].

### 5.1. Gum-Based Nanocomposites

Nanocomposites are composites including at least one phase with nanometer dimension. Nanocomposites can be divided broadly into three classes as: (1) polymer matrix nanocomposites (PMNC); (2) metal matrix nanocomposites (MMNC); and (3) ceramic matrix nanocomposites (CMNC). Nanocomposites are preferred compared to micro- and macro-composites because of their exceptional and potential properties such as optical, mechanical, and barrier characteristics. Nanocomposites involving polymeric matrices and bioactive/resorbable nanofillers have been considered as the proper candidates for nanomedicine and tissue engineering applications. The fillers with nanoscaled size can severely alter the physical traits of the polymeric matrix, permitting the engineering of healed biomaterials that individual materials cannot achieve. The nanomaterials possess an extensive high surface area, compared to conventional microsized fillers, which can generate a strong interface with polymer matrix, suggesting healed mechanical characteristics, while sustaining biocompatibility that affects protein adsorption, cell attachment, proliferation, migration, and differentiation for new tissue formation [109,114].

Nanoparticles, including inorganic nanoparticles (such as clay, HAp, graphene, and metallic nanoparticles), organic/polymeric nanoparticles, and organic/inorganic nanoparticles, can be used as fillers to reinforce the matrix and bring the polymer new functionalities as well [115,116]. For example, Liang et al. designed modern flaxseed gum (FG) nanocomposites for the slow release of iron [117]. After the extraction of FG, the FG–iron nanocomposites were prepared. Then, stimulated gastric fluid (SGF) was used to examine the release of iron from these nanocomposites. The results showed that FG–iron nanocomposites are potential candidates for the slow release of iron in SGF [117]. In another study, Hira et al. fabricated pectin–GuG–zinc oxide nanocomposite (PTN–GuG–ZnO) to increase the lymphocyte toxicity against lung and breast carcinomas [118]. They examined the immunomodulatory activity of PTN–GuG–ZnO on human blood peripheral lymphocytes and showed that the prepared nanocomposite significantly elevates the number and activation of those cells. They also investigated the cytotoxicity of the PTN–GuG–ZnO nanocomposite on A549 and MCF-7 cell lines and demonstrated that this nanocomposite remarkably increased death in these cell lines, and by elevating the E:T ratio from 2.5:1 to 20:1, the number of cell death increased. In addition to the study, a transdermal membrane of poly (N-isopropylacrylamide)–g–GuG nanocomposite reinforced with bio-derived cellulose nanofibril (CNF) has been developed by using free-radical polymerization and the release of diltiazem hydrochloride (DH) from the prepared nanocomposites was investigated during 20 h. The results demonstrated the high release rate in the first 5 h [119]. In another study, Bonifacio et al. designed halloysite nanotube (HNT)-incorporated GeG hydrogels for soft tissue engineering applications [120]. These nanocomposites were biocompatible and safe with adjustable physical properties so that they showed biocompatibility with human dermal fibroblasts. Besides, due to the mechanical and topographical characteristics of HNT content in the hydrogel, the metabolic activity of fibroblasts increased and the highest metabolic activity was observed in 25% content of HNT.

### 5.2. Gums-Based Nanofibrous Forms

Nanofibers have interesting features including extensive surface-area-to-volume ratio and great porosity (i.e., with smaller pore size). Electrospinning and the sol–gel method can be used to make nanofibers from various biodegradable polymers and natural materials. Therefore, nanofibers can be potential materials for numerous biomedical applications including wound dressing, tissue engineering, artificial organ, medical prostheses, drug delivery, pharmaceutics, filtration, and biosensing applications. The electrospun nanofibrous forms are prepared under the uniaxial stretching of a viscoelastic solution due to the electrostatic differences [121]. Recently, the production of nanofibers from natural gums has extensively been studied [122]. Shekarforoushet et al. prepared XG-CS nanofibers for the delivery of bioactive (hydrophobic) molecules [123]. The release of curcumin from these nanofibers for 12 h of analysis in the pH values of 2.2, 6.5, and 7.4 showed no burst effect. However, the release rate of curcumin in pH 2.2 was lower compared to the other pH values.

There is always a need for newly engineered biomaterials with potential characteristics and functional properties for tissue engineering applications. Tamarind gum (TaG) is derived from the endosperm of *Tamarindus indica* Linna. It has an average molecular weight of 880 kDa with a branched structure containing main and side chains. The main chain consists of β-D (1-4)-linked glucopyranosyl units and side chain with a single xylopyranosyl unit linked to every second, third, and fourth D-glucopyranosyl units via α-D (1-6) linkage. TaG is safe (noncarcinogenic) and biocompatible, which results in its extensive applications with diverse pharmaceutical dosage forms [90,124,125,126]. Sangnim et al. formulated clindamycin-incorporated nanofibrous patches composed of polyvinyl alcohol (PVA) and TaG via electro-hydrodynamic atomization [127]. The antibacterial activity of polymeric nanofibrous patches (PNPs) containing 1.0%–2.5% of clindamycin with *Staphylococcus aureus* demonstrated potential bactericidal activity, where the antibacterial activity slightly relies on clindamycin content. GT has been used in foods as a stabilizer, thickener, emulsifier, and texturant additive, and also applied in non-food industries including discoloration of textile waste-water in the form of hydrogel [128] pharmaceutical [35,129] and finishing of textile fabrics [130]. Synthesizing micro- and nano-hydrogels using GT for drug carrier goals [131,132,133,134,135,136] has been investigated more. GT is known for its excellent biological characteristics, including biocompatibility, biodegradability, antibacterial, and wound healing behavior. Electrospinning of pure GT was proved impossible but blending GT with poly (*ε*-caprolactone) (PCL) and or PVA demonstrated the remarkable ability of wound healing via nanofibrous form due to an increase in the collagenation and proliferation steps of the wound repair [137,138,139,140]. These nanofibrous scaffolds showed biocompatibility and antibacterial activity. As described, curcumin-loaded GT/poly (*ε*-caprolactone) electrospun nanofibers have been prepared for diabetic wound healing applications. These nanofibers had antibacterial properties and their results showed that nanofibrous membranes can release curcumin in a prolonged manner and the wound area remarkably was lower in the treated wounds with these scaffolds compared to the control groups in in vivo studies [139]. Almond gum (AG) is a natural, colorless, odorless, and non-toxic polysaccharide, which, in its dry state, has proteins (2.45%), fats (0.85%), and carbohydrates (92.36%). In this case, carbohydrates include arabinose (46.83%), galactose (35.49%), uronic acid (5.97%), with slight levels of rhamnose, mannose, and glucose [141]. Besides, gum exudate comprises different minerals (i.e., sodium, potassium, magnesium, calcium, and iron) [142]. AG has high molecular weight and is extracted from the branches, trunks, and fruits of almond tress [143]. AG has an arabinogalactan structure and contains water-soluble and water-insoluble fractions. It has been reported that almond gum is a better emulsifier compared to the gum arabic (ArG) [144]. Because of its antioxidant and antimicrobial characters, it can elevate the biological and functional properties in various food formulations [145]. Rezai et al. prepared AG/PVA nanofibers for the delivery of vanillin [146]. The incorporation of vanillin in AG/PVA nanofibers significantly increased the thermal stability compared to vanillin-free nanofibers, making these nanofibers thermally stable delivery system for vanillin.

**Figure 2:**
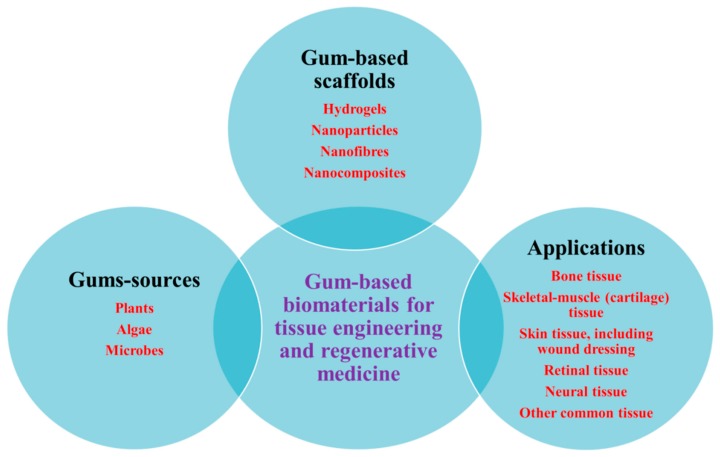
Schematic drawing of gums sources, gums-based scaffolds and scaffold components, and their potential tissue engineering applications.

XG was first discovered in the 1950s. XG is a high molecular weight polysaccharide with branched polymeric chains, great water-solubility, and excellent biocompatibility [147,148]. XG consists of D-glucosyl, D-mannosyl, D-glucuronyl acid, O-acetyl, and pyruvyl residues. XG and XG-derived biomaterials have been applied in food, food packaging, cosmetics, tissue engineering, drug delivery, water-based paints, water treatments, jet injection printing, oil recovery, petroleum industry, construction, and building materials [89]. Furthermore, XG nanofibers can be used for the delivery of hydrophobic bioactives [123]. A schematic representation of the natural gum sources, gums-based scaffolds, and scaffold components, and their potential tissue engineering applications is shown in Figure 2.

## 6. Natural Gums as Biocompatible Scaffolds for 3D Cell Culture and Tissue Engineering

Hydrogels are described as a water-swollen 3D network, where a native 3D microenvironment is needed cell fate and as promising candidates for further development of new tissue or complex tissue regeneration [149] due to their structural similarity to the ECM of the native tissues [150]. In this case, hydrogels can easily mimic the tissue microenvironment with tunable mechanical properties to optimize the regeneration of tissues in vivo and formation of tissues in vitro [151]. In tissue engineering applications, polysaccharides have potentially been used for the development of hydrogels due to their different chemical, physical properties, and biological activity [152]. 

Recently, natural gums in different formulations and designs have widely been applied in different tissue engineering applications to provide the possibly correct 3D microenvironment to promote the accurate in vitro and in vivo native tissue formation [149,153,154,155]. 

### 6.1. Bone Tissue Engineering

To improve the regeneration of bone tissues, XG–HAp composite hydrogels have been prepared by the mineralization of HAp using the alternate soaking method. In this study, the results showed a variation in the microstructure from layered to porous microstructure in the gel system. The increased soaking cycles reduced the mechanical performance, whereas the mechanical stability remained high even after 5 or 10 cycles by allowing the elongation of 380% and 200%, respectively [44]. In another study, ArG/HAp scaffolds exhibited favorable biocompatibility, a similar structure to native bone (macroporosity: 87%–93%, average pore size: 164–230 μm), where ArG/HAp50 scaffolds showed ultimate compressive strength (~16.6 MPa), the highest compressive modulus (~75.3 MPa), and increased alkaline phosphatase (ALP) levels and calcium deposition when cultured with C2C12 cells [156]. Further, the incorporation of HAp in GeG-based spongy-like hydrogels exhibited high swelling, sustained degradation, pore size (200–300 μm), and higher porosity (>90%). Both Ca^2+^ and HAp improved the mechanical stability of the hydrogels (storage modulus: 40–80 kPa). In addition, hydrogels showed good bioactivity and bone cell attachment and spreading within the hydrogels up to 21 days of cell culture [157]. Further, ALP, an enzyme that is involved in the biomineralization of bone by cleaving phosphate from organic phosphate, was incorporated in GeG hydrogels to induce biomineralization with calcium phosphate (CaP). In this case, ALP facilitated the formation of submicron-scaled ‘apatite-like’ material within GeG hydrogels and showed an increased amount of CaP including stiffness with increased ALP content. However, the mineralized GeG hydrogels were found to be stable under sterilization by autoclaving, while stiffness was observed to be decreased. In addition, the incorporation of polydopamine (PDA) improved the mineralizability and stiffness of GeG hydrogels. Moreover, this mineralization of GeG hydrogels led to improved cell attachment and viability in vitro [158]. Further, these ALP-mediated GeG hydrogels were mineralized by incubation in media containing calcium glycerophosphate (CaGP) and magnesium glycerophosphate (MgGP). Here, various mineralization media with CaGP:MgGP concentrations (mol/dm^3^) 0.1:0 (A), 0.075:0.025 (B), 0.05:0.05 (C), 0.025:0.075 (D), and 0:0.1 (E) were compared and results showed the incorporation of higher Ca content into mineral than Mg content, when samples were mineralized in medias A–D. The amorphicity of the formed minerals as well as Mg content increased in the order A < B < C < D. The mineral formed in medias A and B was calcium deficient HAp, while there was a combination of calcium deficient HAp and an amorphous phase in media C. Further, only an amorphous phase was observed in the mineral formed in media D, whereas a combination of crystalline and an amorphous phase was observed in the mineral formed in media E. The Young’s and storage modulus of the samples mineralized in the media were decreased in the order A > B > C > D, while increased significantly for samples mineralized in media E. Furthermore, the osteoblastic MC3T3-E1 cell attachment and viability was higher on samples mineralized in medias B–E (having Mg) compared to those mineralized in media A (not having Mg). All samples showed degradation and supported the attachment of RAW 264.7 monocytic cells and samples also supported osteoblast-like cell formation when mineralized in medias A and B [159]. 

**Figure 3:**
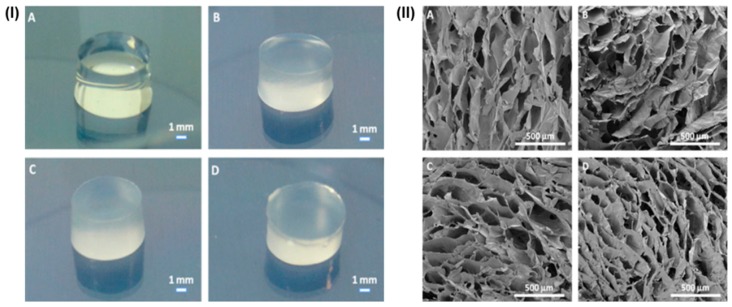
Digital images (**I**) and SEM images (**II**) of GG-based hydrogel disks: non-crosslinked GG (**A**); ionic crosslinked GGMA disks (**B**); MBF (0.1% *w*/*v*) photocrosslinked GGMA disks at 366 nm (**C**); and HHMPP (0.05% *w*/*v*) photocrosslinked GGMA disks at 240–300 nm (**D**). Reproduced with the permission from [162]. Copyright 2010, Elsevier.

Apart from HAp, bioactive glass (BG) also exhibited a potential impact in hard tissue regeneration. In this advancement, Ca^2+^ ions crosslinked GeG/bioactive glass (BG) hydrogels have been developed for bone tissue regeneration, where three types of BGs—one Ca-rich (A2), the second Ca-deficient (S2), and the third closely mimicking the commonly used 45S5 type BG (NBG)—were prepared and compared. The results showed good apatite formation in the case of BG-containing hydrogels (not in BG-free hydrogels) when incubated in simulated body fluid (SBF). Further, the enhanced compressive modulus was observed in hydrogels with S2 or NBG, but not in A2. Higher antibacterial activity was analyzed in hydrogels with A2 and S2 compared to NBG-reinforced and BG-free hydrogels. Furthermore, a stimulatory effect of NBG on rat mesenchymal stem cells (rMSCs) differentiation was observed [160]. Further, the incorporation of BG NPs facilitates apatite formation ability (in vitro) when soaked in simulated body fluid (SBF) and showed good human adipose-derived stem cell attachment and spreading within the BG/GeG spongy-like hydrogels and remain viable. In addition, GeG/BG spongy-like hydrogels showed an improved microstructure with an agglomeration of globular BG NPs within the GeG hydrogel network. The results exhibited only Young’s modulus of 1.9 MPa (in dried form) and 1.2 MPa (in hydrogel form) were measured for the BG/GeG (50:50) hydrogels [161]. In another study, ionic and photo crosslinked GeGMA hydrogels, for intervertebral discs (IVDs) regeneration (see Figure 3), showed enhanced mechanical performances, low water uptake capacity, and low degradation behavior compared to pure GeG hydrogels. In addition, these hydrogels exhibited good cytocompatibility in vitro when cultured with rat lung fibroblast L929 cells [162]. 

As compared to HAp and BG, HNTs also improved the mechanical properties and biocompatibility of the hydrogel networks. Physically crosslinked GeG/glycerol (Gly) hydrogels reinforced with HNTs showed the well-improved biocompatibility of the GeG-Gly hydrogel. The incorporation of HNTs led to a decrease in water uptake (30%–35%) and tunable compressive Young’s modulus (20–75 kPa). Moreover, GeG-Gly/HNTs (25%) hydrogels exhibited high metabolic activity and cell viability of human dermal fibroblasts up to seven days of culture [120]. 

Good injectability, mechanical stability, and good cytocompatibility of CCG hydrogels reinforced with whitlockite NPs and dimethyloxallylglycine (an angiogenic drug) with human umbilical vein endothelial cells were observed. Overall, CCG hydrogels promoted osteogenesis and angiogenesis in vitro [63]. In another study, the addition of 0.5% GuG improved the hydrogen bonding of soybean protein isolate (SPI) adhesives with a maximal bond strength of 5% SPI adhesive and that was 2.8-fold higher than those of control. The loosened adhesives formed an amorphous structure with a stronger glue network that facilitates the bonding between adhesives and bones [163]. Further, compared to tissue culture plate (TCPS) and collagen hydrogel, GT hydrogel exhibited the highest mineralization and ALP activity with adipose-derived MSCs (ADMSCs). Relative gene expression levels exhibited the highest expression of Runx2, osteonectin, and osteocalcin in GT hydrogel cultured with cells, but the expression of collagen type I remains constant [164]. The incorporation of cellulose nanocrystals (CNCs) in XG/silica glass (SG) scaffolds showed tunable and improved mechanical properties with good pre-osteoblast MC3T3-E1 cells cytocompatibility [165]. In addition, the effect of variable amounts of CNCs and/or HNTs on sodium alginate (SA)/XG (SAX) scaffolds also showed improved thermal stability, mechanical properties (under compression), and in vitro cytocompatibility with MC3T3-E1 osteoblastic cells as compared to SA and SAX scaffolds [166]. By the co-precipitation method, Bael fruit gum (BFG)–CS/HAp nanocomposite scaffolds have been prepared. The addition of BFG in the nanocomposite scaffold showed significant improvement in compressive strength and modulus, swelling behavior, biodegradation, protein adsorption, and antibacterial activity. Further, in vitro bioactivity was demonstrated by the formation of a thick apatite layer and improved cell attachment, proliferation, and osteogenic differentiation was observed [167].

### 6.2. Osteoarthritis and Cartilage Tissue Engineering

**Figure 4:**
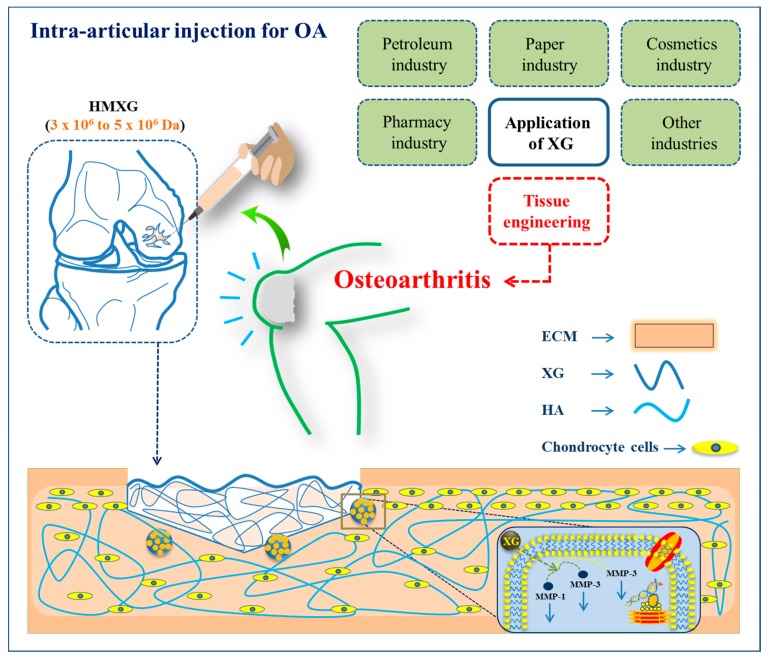
Schematic representation of various industrial applications along with tissue engineering application of xanthan gum (XG) and the preparation of HWXG intra-articular injection for osteoarthritis therapy by suggesting mechanism [173].

Osteoarthritis (OA) is one of the most common chronic diseases and evaluated by the degradation of articular cartilage. Therefore, the efficacy of intra-articular injection of allogeneic ADMSCs combined with XG in rat osteoarthritis (OA) model has been investigated. In this study, XG improved in vitro proliferation of ADMSCs significantly in a dose-dependent manner and a single intra-articular injection of allogeneic ADMSCs combined with XG efficiently lessened OA progression with a therapeutic effect, which was significantly higher than that of either XG or ADMSCs alone [168]. Further, the intra-articular injection of XG once every two weeks up to five weeks, compared to the negative control group, inhibited chondrocytes apoptosis, matrix metalloproteinase-1, -3 protein expression significantly, and also improved the tissue inhibitors of matrix metalloproteinase-1production in cartilage [169]. In another study, XG could reverse sodium nitroprusside (SNP)-reduced cell proliferation significantly and inhibited early cell apoptosis rate in a dose-dependent manner. In addition, XG reduced the loss/disruption of mitochondrial membrane potential and decreased PGE2 level of cell culture supernatants in SNP-induced chondrocytes [170]. As described above, XG injection with high molecular weights (Mw) (HMXG; 3 × 10^6^–5 × 10^6^ Da) could improve the viscosity of the synovial fluid that protects joint cartilage in rabbits. However, there was no significant difference between the existing clinical medication (sodium hyaluronate, HA) and the obtained XG injection at the same frequency (once weekly for five weeks; see Figure 4) [171,172]. Further, an injection of XG with a low molecular weight (LWXG; 1 × 10^6^ Da to 1.5 × 10^6^ Da) for a long-acting drug for treating OA has been compared at once every two weeks up to five weeks as with HA at once weekly up to five weeks as a reference.

In this case, LWXG also could protect cartilage tissue from damage, reduce the concentration of nitric oxide (NO) in the synovial fluid, and is able to reverse the amplification of knee joint width similar to high molecular weight (HMXG) [169,172]. LWXG promotes chondrocytes proliferation while decreasing apoptosis at the cellular level. These effects are evoked via down-regulation of the levels of protein of caspases-3 and bax and up-regulation of levels of protein of bcl-2 in cartilage both in vitro and in vivo [173]. As more adhesion between the surgical tendon and the synovial sheath after tendon-repair surgery shows a poor functional repairing of the tendon. In this case, the developed certain XG–GeG–HA membranes showed equal reduction ability of the adhesion of tendons, without decreasing the tendon strength as compared to the commercially available Seprafilm. Moreover, these membranes showed slow degradation that allows functioning as barriers for longer periods [174].

Shin, Olsen, and Khademhosseini prepared double-network (DN) hydrogels via a two-step photocrosslinking using GeG-methacrylate (GeGMA) for a rigid and brittle first network and gelatin-methacrylate (GelMA) for a soft and ductile second network for the possible encapsulation of cells. The obtained GeGMA/GelMA DN hydrogels showed compressive failure stress up to 6.9 MPa that approaches the cartilage strength. In this case, a higher mass ratio of GelMA to GeGMA in DN hydrogels showed higher strength and exhibited good cell compatibility by encapsulating NIH3T3 fibroblast cells and their viability [175]. Further, GeG hydrogel disks showed a compressive storage and loss modulus of ~40 kPa and 3 kPa at 1 Hz frequency, respectively. Further, a rheological analysis showed a sol–gel transition that started at 36 °C with a gelation time of 11 s. In addition, hydrogel exhibited good cell viability with human nasal chondrocytes (1 × 10^6^ cells/mL) when cultured for a total periods of two weeks [176]. The application of GeG in cartilage tissue engineering is limited due to the high gelation temperature [177] and lack of mechanical properties. Therefore, the use of carboxymethyl-CS (CMCS) with oxidized-GeG double-network hydrogel (O-GeG) improved the gelation temperature and mechanical properties compared to pure GeG (P-GeG) hydrogel. In this study, gelation temperature was lowered from 42 °C to below physiological temperature by oxidation and subsequently reduced by complexion with CMCS. The hydrogel exhibited improved compressive modulus (278 kPa) and good shape-recovery when the load was released while showing significant improvement in cell viability with chondrocytes. Moreover, P-GeG and complex-GeG (C-GeG-3) exhibited lower cytotoxicity compared to oxidized-GeG (O-GeG-3) and subsequently cell proliferation on P-GeG and C-GeG-3 was greater than on O-GeG-3, where 3 denotes to the dosage of NaIO_4_ (0.05 M) in mL [178]. 

Perugini et al. designed and developed ionically crosslinked methacrylated GeG (iGeG-MA) hydrogel disks and subsequently bio-functionalized with FF-Gen_3_K(WHLPFKC)_16_ to improve the clinical performance of cartilage tissue regeneration. The dendronized structures at nanoscale concentrations were able to improve the control on the biological behavior of WHLPFKC at the tissue/material interface. Moreover, the in vivo analysis showed that the dendron VEGF blockers functionalized iGeG-MA the inhibition of angiogenesis by controlling both the size and ramifications of blood vessels in the proximity of hydrogel boundary implanted at the targeted site [179]. Further, photo-crosslinkable methacrylated GeG (GeG-MA)/polyethylene glycol dimethacrylate (PEG-DMA) hydrogels showed better mechanical performance compared to only GeG-MA hydrogel, although not suitable enough for injecting at the site of damaged soft tissues. Moreover, the obtained injectable hydrogels showed good cytocompatibility with human fibroblast cells (WI-38 cells) [180]. Further, the gelling parameters of GeG have been optimized for fibrocartilage tissue engineering. the increased amount of low acyl-GeG resulted in improved stiffness and the incorporation of high acyl-GeG exhibited a high decrease in stiffness. Therefore, it was concluded that 2% (*w*/*v*) low acyl-GeG would have the most appropriate gelling temperature, degradation, and mechanics to be used in fibrocartilage tissue engineering [181]. In another study, the potential role of miR-30a to assess the possible differentiation of chondrogenic combined with SF/GeG hydrogels for sustainable cell viability and proliferation has been demonstrated and positively affected the cartilage regeneration, and can promisingly be used as therapeutics for osteoarthritis [182]. For OC regeneration, GeG/Hap-based bilayered hydrogel constructs showed good cytocompatibility with rabbits’ chondrocytes and osteoblasts encapsulated in respective layers, and OC tissue was successfully regenerated after four weeks after implantation in vivo (mice) [183]. 

### 6.3. Skin Tissue Engineering

Significant advances have been achieved in preparing skin substitutes, including wound healing over a long time. In this case, electrospun hybrid nanofibers composed of CS–gelatin–PVA–ArG with 8:8:2:0.5 (C8G8P2A0.5) weight ratios showed good stability and tensile strength (2.53 MPa) and excellent MSCs (called as KP-hMSCs) attachment and proliferation [184]. In another study, electrospun scaffolds composed of PCL (for elasticity and strength), Zein (as corn protein), and GA (as polysaccharide) have been prepared and the PCL/Zein/GA scaffolds showed highly fibrous structure with high hydrophilicity and mechanical properties (tensile strength: 1.36–3.0 MPa and elongation: 19.13%–44.06%), which are desirable for the engineering of skin tissues. Additionally, GA-incorporated PCL/Zein scaffolds exhibited good antibacterial activities and favorable L929 cell proliferation [185]. In addition to this study, *Calendula officinalis* (*C. officinalis*) extract has been incorporated in PCL/Zein/GA nanofibrous scaffolds which were prepared in three ways: (1) by suspension electrospinning (PCL/Zein/GA/*C. officinalis* layers); (2) by two-nozzle electrospinning (PCL/Zein/GA and PCL/*C. officinalis* layers); and (3) multilayer electrospinning (PCL/Zein/GA and PCL/*C. officinalis* layers). PCL/Zein/GA/*C. officinalis* scaffolds showed suitable mechanical properties and desired degradation behavior for the engineering of skin tissues. However, multilayered PCL/Zein/GA/*C. officinalis* scaffolds exhibited higher tensile strength as compared to other nanofibrous scaffolds. PCL/Zein/GA/*C. officinalis* scaffolds demonstrated favorable fibroblast cell attachment and proliferation, and antibacterial activities compared to PCL/Zein/GA scaffolds [186].

#### Wound Dressing

Acacia gum (AcG)/polyvinylpyrollidone/carbopol hydrogel films for wound dressing exhibited antioxidant, non-hemolytic, and mucoadhesive in nature and absorbed wound fluid. In addition, hydrogel films that showed the release of the moxifloxacin drug from the hydrogel network followed non-Fickian diffusion mechanism and were best-fitted to the Higuchi model [187]. Further, ZnO NPs show antimicrobial behavior and improve the wound healing process. In crosslinked SA–AcG/ZnO hydrogels, ZnO NPs concentration-dependent zones of inhibition (antibacterial effect) against an antibiotic-resistant *Pseudomonas aeruginosa* and *Bacillus cereus*, and cytocompatibility analysis on peripheral blood mononuclear/skin fibroblast cells were demonstrated. The low concentration of ZnO NPs in the SA–AcG matrix showed a good healing effect using sheep fibroblast cells, while a high concentration of ZnO NPs was observed to be toxic to skin fibroblast cells. However, the obtained hydrogels reduced the toxicity significantly, while preserving healing and antibacterial effect [188]. 

**Figure 5:**
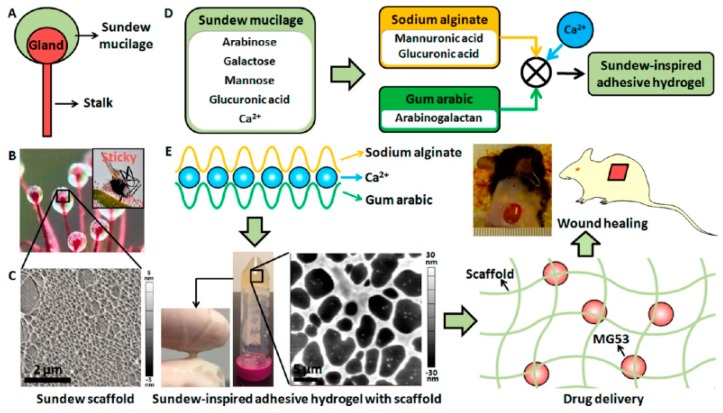
(**A**) Schematic diagram and (**B**) actual image of Sundew-inspired adhesive hydrogel (mucilage) for controlled drug delivery in a chronic wound healing. Inset in (B) exhibits a prey trapped by the Sundew mucilage. (**C**) AFM image of the Sundew mucilage; (**D**) SA, ArG, and Ca^2+^ used for Sundew mucilage; and (**E**) hydrogel forming by SA and ArG with Ca^2+^ as crosslinking agent, characteristic of adhesive hydrogel by touching and pulling the hydrogel, porous network structure (AFM image), and the potential applications for drug delivery and wound healing. Reproduced with the permission from [190]. Copyright 2017, American Chemical Society.

Bouaziz et al. generated AG oligosaccharides (galactose and arabinose, including the traces of glucose, xylose, mannose, and rhamnose) by enzymatic hydrolysis and the effect of as-generated oligosaccharides on full-thickness wound of rats (on the dorsum of rats) either alone or supplemented with cream formulation as an active substance showed wound closure (%) as an average of around 100%, while at the same day, the healing (%) of the control group was observed only 74.3%. An improved collagen deposition along with increased fibroblast and vascular densities were observed [189]. In another study, a sundew-inspired SA hydrogel to deliver mitsugumin 53 (MG53; an important protein in the repair of cell membranes) for chronic wound healing was developed and showed in vitro biphasic kinetics release by facilitating both the fast delivery of MG53 to improve the re-epithelization process and sustained release of the protein to treat chronic wounds. Further, in vivo analysis demonstrated enhanced dermal wound healing in the mouse model, where sundew-inspired hydrogels were encapsulated with rhMG53 cells. The schematic formation and mechanism of sundew-inspired adhesive hydrogel for controlled drug delivery and chronic wound healing is shown in Figure 5 [190].

Moreira et al. developed a bioactive cashew gum (CG)/PVA wound material (film) that facilitated the modulate proteinases activity and simultaneously prevented infection by blocking out pathogenic microorganisms and other foreign species. In this study, the activated film by the covalent immobilization of trypsin remained 100% active after storing for 28 days and drying at room temperature and even could be used for nine cycles of use or storage without activity loss (collagenolytic). The obtained wound material films showed excellent human PDL fibroblast cell attachment and viability when cultured on the surface of the films [191]. The developed Apigenin (APN) loaded-GeG/CS hydrogel showed 87.15 ± 1.20 entrapment efficiency (%) and released 96.11% APN in 24 h. Further, the level of superoxide dismutase (SOD) and catalase was significantly improved in granuloma tissue of the APN-treated group. APN–GeG–CS hydrogels showed a higher healing effect in diabetic as well as normal wound tissues with significant antioxidant activity [192]. Further, GeG/HA spongy-like hydrogels accelerated wound closure rate, re-epithelization, and tissue neovascularization upon transplantation into full-thickness mice wounds. At the early stages, the synergistic effect of the GeG/HA hydrogel and the transplanted cells over those processes was investigated. Despite human-derived and chimeric blood vessels found, the obtained spongy-like hydrogel did not succeed in prolonging the time of cell residence and sustained self-organization of the transplanted human cells possibly due to primitive degradation [193]. However, these cell-adhesive GeG/HA spongy-like hydrogels demonstrated a successful integration of prevascularization cues of hydrogel to target angiogenesis/neovascularization in skin full-thickness excisional wounds [194].

The crosslinked water-insoluble GeG films (GeG40) (thickness 26 μm) exhibited high gel content (73%), tensile strength (52.4 MPa), good cytocompatibility behavior with L929 cells, and inhibited the platelets’ absorption and activation. In addition, GeG40 film showed slight inflammation in rat subcutaneous tissue at the early postoperative duration, but no fibrosis or stromal reaction was observed in either short-duration or long-duration implantation [195]. Horii et al. investigated the effect of partially-hydrolyzed-GuG (PHGuG) on wound healing via PHGuG-mediated activation of RhoA, which is dependent on ERK1/2 activation, to improve the colonic epithelial cell wound healing. In this study, PHGuG treated with young adult mouse colonic (YAMC) epithelial cells significantly improved the healing of wounds as compared to control. However, this improvement was inhibited by both Y-27632 (RhoA inhibitor) and U0126 (ERK1/2 inhibitor). PHGuG-dependent improvement was observed in the accumulation of F-actin and Rho kinase activity (blocked by U0126), while PHGuG-dependent ERK1/2 activity was not inhibited by Y-27632 [196]. The ceftazidime drug possesses a broad range of antibiotic activity and additionally could be used to provide antimicrobial behavior. For this, aminated carboxymethyl-GuG (CMGuG) crosslinked with fish collagen by ceftazidime drug via ionic interactions has been developed. In this study, the crosslinked film showed 90%–95% Ceftazidime drug release from the aminated CMGuG Ceftazidime drug collagen (ACCC) film at a physiological pH after 96 h of incubation. In addition, good cytocompatibility and blood compatibilitym as well as good growth inhibition against *S. aureus* and *P. aeruginosa*, was observed [197]. 

GT/PVA electrospun mats showed good antibacterial property against Gram-negative bacteria and good cell attachment and proliferation when cultured with human fibroblast AGO cells [137]. Further, the developed GT/PCL nanofibers as patches for the healing of diabetic wounds have been demonstrated as promising vehicles for the sustained and efficient delivery of 3% curcumin (Cur) up to duration of 20 days and exhibited enhanced mechanical performance (stability of scaffold in front of blood and fibrin) and cell attachment and proliferation compared to GT/PCL nanofibers [138]. In addition, PCL/GT/Cur nanofibrous membranes showed fast and significant wound closure with well-formed granulation tissue dominated by the proliferation of fibroblast and deposition of collagen followed by the complete early regenerated epithelial layer and the development of sweat glands and hair follicles, compared to control [139]. In another study, the same authors developed GT/PCL/PVA nanofibrous scaffolds and demonstrated the favorable mechanical performance, biocompatibility, antibacterial behavior, as well as their hydrophilic nature for skin tissue engineering applications [140]. The composition PVA-*cl*-GT-*co*-SA-based wound dressing films exhibited good absorption of wound fluid and showed blood-compatibility and non-thrombogenic nature. The obtained wound dressing films showed good gas permeability, impermeability to microorganisms, and controlled release of moxifloxacin drug for 24 h of time duration without burst release [198]. Further, Aloe Vera extract encapsulated and aluminum ions (Al^3+^) crosslinked GT spherical-shaped nanocapsules showed relative great antimicrobial behaviors as 84%, 91%, and 80% reduction against *Escherichia coli*, *S. aureus*, and *Candida albicans*, respectively. In addition, these wound products exhibited 98% cell viability of human fibroblast cells and showed good wound healing behavior by involving the significant rate of migration of the cells [199]. Silver NPs (AgNPs)-incorporated GT hydrogels showed excellent antibacterial behavior against *E. coli* (Gram-negative) and *Bacillus subtilis* (Gram-positive) [200]. Polyelectrolyte complex (PEC) hydrogels have shown great potential in the biomedical area [201,202], especially tissue engineering applications. In this case, Chlorhexidine (CHX)-loaded XG-CS PEC microspheres showed good cytocompatibility with human fibroblast cells and selective antibacterial activity (i.e., *Porphyromonas gingivalis*) as measured by blood-sugar plate assay [203]. 

### 6.4. Retinal Tissue Engineering

The retina is composed of very fine and layered neural tissues and retinal degeneration has been a major challenge worldwide [204]. Several biomaterials have been developed and research is still going on to achieve proper treatment. In this advancement, polyethylene glycol (PEG)/GeG hydrogels have been developed for retinal regeneration. In this study, the effect of PEG amounts was investigated on ARPE-19 cell attachment and proliferation in PEG/GeG hydrogel. PEG/GeG hydrogels showed superior biocompatibility (>90%) via cell attachment and proliferation and a positive impact on RPE-specific gene expression as compared to only GeG hydrogels [205]. 

### 6.5. Neural Tissue Engineering

Regeneration of nerves is a complex phenomenon of the biological system, where small injuries can be regenerated on their own, but large injuries need proper surgical treatment [206,207,208,209]. For large nerve repair, several approaches have been investigated for peripheral nerve regeneration and repairing spinal cord [206]. In this advancement, bioamine (spermidine (SPD) or spermine (SPM))-crosslinked GeG hydrogels allowed the encapsulation and migration of human neurons in the GeG hydrogel network. Under physiologically relevant stress and strain, the obtained hydrogels resembled closely or showed bio-mimicking comparable to native rabbit brain tissue and showed good cytocompatibility with human pluripotent stem cell-derived neuronal cells. In addition, laminin functionalized GeG hydrogels exhibited cell-type-specific behavior in neuronal cell maturation and neurite migration. In this case, GeG hydrogel with 3.0% SPD is considered more supportive of the formation of the 3D neuronal network within the hydrogel to be used in neural tissue engineering [210].

**Figure 6:**
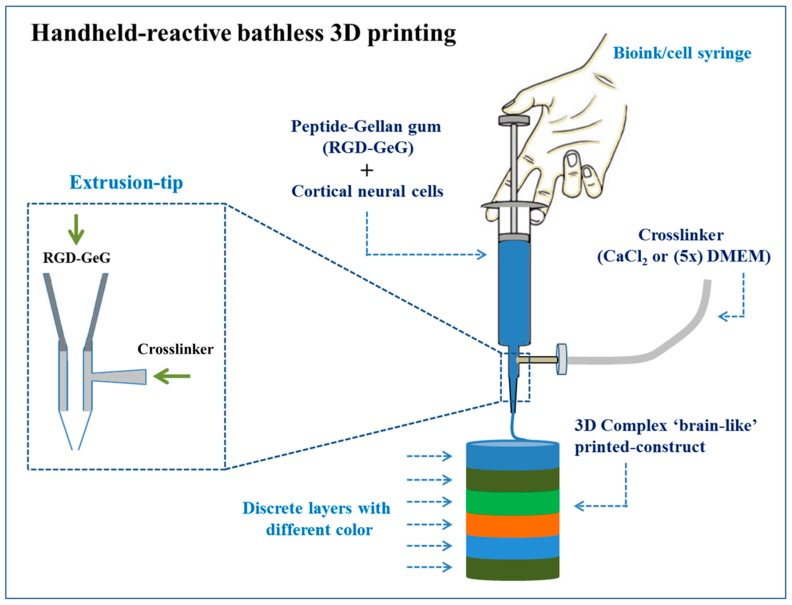
Handheld reactive bathless 3D printing: Schematic diagram of extrusion-tip and 3D printed ‘brain-like’ layered-construct composed of peptide (RGD), gellan gum (GeG), and cortical neural cells (i.e., each color denoted by a layer) [211].

The brain is a complex organ system and therefore the development of in vitro model accurately for the brain remains an obstacle significantly for the understanding of functions of the brain at the level of tissue or organ. Lozano et al. developed a method to form 3D complex ‘brain-like’ constructs with discrete layers of encapsulated primary cortical neural cells. In this case, the bio-ink for the printing of brain-like structures was composed of GeG, peptide (RGD), and primary cortical neurons. The peptide modification of GeG hydrogels was observed to have an intense and positive effect on cell proliferation and network formation, demonstrating the cells’ supportive nature of the hydrogel network and the formation of the cell-network in specific layered-constructs. The fully assembled system of handheld reactive bathless 3D printing and a manual extrusion of bio-ink from syringe produced a solid hydrogel construct (see Figure 6) with precise replication. 

The printing of a structure (six-layered) by using 0.5% *w*/*v* RGD-GeG with various dyes in each printed layer was performed. Here, printed-structure was processed with a top-layer (0.5 *w*/*v* RGD-GeG with 1 × 10^6^ cells/mL), a middle-layer (no cells), and a bottom layer (0.5 *w*/*v* RGD-GeG with 1 × 10^6^ cells/mL). After five days of culture, the cell formed a neuronal network and axons began to infiltrate in the acellular middle-layer [211]. In addition, aligned poly (L-lactic acid) (PLLA)/GT 75:25 as nanofibrous mats with balanced properties showed good nerve cell (PC12) attachment, proliferation, enhanced bipolar neurite outgrowth, better cell differentiation and phenotype, and nerve cell orientation along the fiber alignment, while random PLLA/GT 75:25 nanofibrous mats showed a 20% decrease in cell proliferation after eight days of in vitro PC12 cells culture [212]. Further, programmable anisotropic 3D structures of methacrylated GeG-based matrix with precise pore diameters have been developed as 3D-oriented neural tissue models. In this case, tunable microporosity is capable to direct cellular responses at millimeter scale and further this process was integrated with a microfluidic system to establish a neural endothelial heterotypic conjugation for promising application in multiple organ systems [213].

### 6.6. Other Tissue Engineering

GeG–graphene oxide (GO) composites are directed to self-assembling into aligned nacre-like (brick-and-mortar microstructure) structure to prepare strong and tough bioinspired composite films showing fracture strength (88.7 MPa), fracture strain (0.84%), and tensile modulus (25.4 GPa), and good cytocompatibility that are comparable to natural nacre [214]. Further, tunable methacrylated GeG (MAGeG) hydrogels, by involving both physical crosslinking (temperature and addition of cations) and chemical (via photocrosslinking) crosslinking, showed the values of Young’s modulus (0.15–148 kPa) and exhibited good in vitro biocompatibility with NIH-3T3 fibroblast cells [177]. Further, cell-adhesive peptides induced GeG spongy-like hydrogels with improved mechanical properties have been developed to promote attachment and spreading of human adipose stem cells (hASCs), dermal microvascular endothelial cells (hDMECs), and human adult skin keratinocytes (hKC), and human osteoblast-like cells (SaOs-2). These hydrogels allowed the cell entrapment and spreading of mesenchymal, epidermal, and osteoblastic phenotypes [215]. In addition to this, GeG-based spongy-like hydrogel modified with polypyrrole (PPy) showed a good conductive system for skeletal muscle tissue engineering and good electrical stimuli effects on skeletal cells [216]. In another study, 3D printing of GeG has been investigated for wound dressing and cartilage tissue engineering by creating complex constructs, and 3D printed-constructs with high surface area to mass ratio facilitate high degradation rate, while compressive strength and modulus increased after degradation in SBF [217]. Uniform and randomly oriented PVA–GeG nanofibrous scaffold demonstrated the non-toxicity and supported the growth of murine embryonic stem cells (ESCs) in scaffolds that make it a potential biomaterial for various tissue engineering applications [218]. 

**Figure 7:**
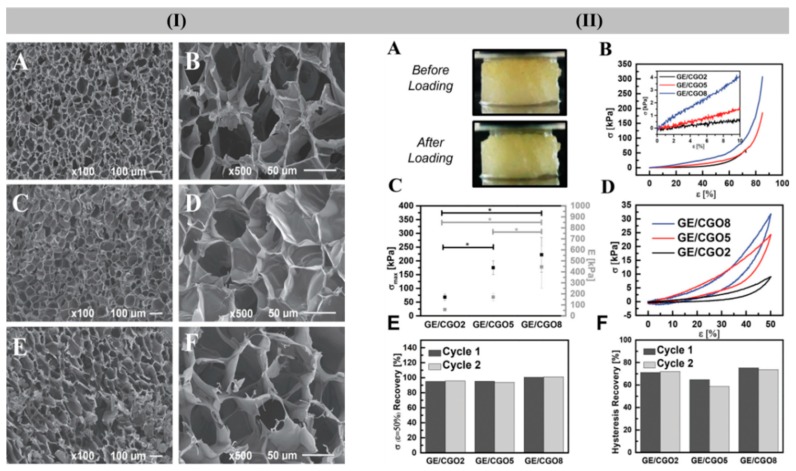
(**I**) SEM images of freeze-dried GE/CGO2 (**A**,**B**); GE/CGO5 (**C**,**D**); and GE/CGO8 (**E**,**F**) sponges at scale bar of 100 μm and 50 μm, respectively. (**II**) Mechanical analysis: (**A**) digital images of GE/CGO8 hydrogels before and after compression (until 90% strain); (**B**) stress–strain curves of GE/CGO hydrated hydrogels (inset: magnified region (0–10% of strain) for measuring compressive modulus (**E**); (**C**) maximum compressive stress (σ_max_) and modulus (**E**) of GE/CGO sponges; (**D**) loading–unloading cycles of GE/CGO sponges until 50% of strain, and (**E**,**F**) recovery of the compressive stress at 50% of strain and hysteresis upon three successive loading–unloading cycles, respectively. Reproduced with the permission from [7]. Copyright 2019, John Wiley and Sons.

For vascular tissues, multi-material anatomically-shaped tissue constructs (hydrogel-tubes) have been prepared by using GelMA, GeG, PCL, and alginate. In this case, the GelMA-GeG hydrogel tube was reinforced with PCL fibers and alginate as the supportive matrix. The incorporation of GeG improved the shape-fidelity of GelMA hydrogel without affecting cell viability and further PCL and alginate were removed from the hydrogel tube, and injected BMSCs-laden gelatin hydrogel into produced open lumen into GelMA-GeG hydrogel tube [219]. As high phase transition of GeG inhibits its applicability in tissue engineering, GeG has been functionalized with methacrylic anhydride to reduce its phase transition temperature. Further, BMSCs-encapsulated hydrogel based on functionalized GeG and type I collagen was prepared for proper vascularization by providing excellent 3D microenvironment and promoting differentiation into endothelial cells [220]. Maciel et al. developed crosslinked hydrogel sponges composed of oxidized CG and gelatin. These hydrogels maintained high absorption of water and recovery of their initial mechanical properties under cyclic-compression (see Figure 7). The obtained mechanically-robust hydrogels might have great potential in supporting cell attachment and proliferation in soft tissue engineering applications [7]. 

PEC hydrogels facilitate the counterions located within the hydrogel network that respond to electrically-induced chemo-mechanical contraction under biological responses. In this way, XG-CS/HNTs PECHs have been prepared in the presence of a green acidifying agent. The obtained PECHs showed good osteoblastic (MC3T3-E1) cell viability and proliferation with an increased amount of HNTs [221]. Further, tunable magnetically-responsive XG-CS/magnetic NPs (Fe_3_O_4_ MNPs)-based PEC hydrogels showed significantly improved mechanical performance, storage modulus (G’), thermal properties, improved fibroblast (NIH3T3) cell attachment, and growth under an external magnetic field compared to PEC hydrogels without MNPs [222]. In another study, PEC hydrogels have been prepared by using sodium carboxymethyl cellulose (CMC), CS, HNTs, and GO. In this study, the effect of HNTs and/or GO with varying content was investigated on the structure–property relationship of CMC-CS PECHs. The results showed the synergistic effect of both HNTs and GO on physico-mechanical and biological properties of PECHs [223]. For soft tissue engineering, ionically crosslinked CCG-XG hydrogels have been prepared by incorporating HNTs and/or carboxylated-CNCs (*c*CNCs). CCG-XG/HNTs (20 wt.%)/cCNCs (20 wt.%) hydrogel showed enhancement in compressive strength (8.1 ± 1.35 kPa) compared to CCG and CCG-XG with or without HNTs. In addition, good cell adhesion and proliferation of human skin fibroblast cells (CCD-986Sk) on hydrogels was observed for seven and 14 days of incubation. In this study, HNTs and cCNCs had a synergistic effect on CCG-XG based hydrogels [61]. In another study, a series of GuG (molecular weight ranging from 74 to 210 kDa)/glycidyl methacrylate (GMA) hydrogels showed linearly decreased enzymatic degradation in vitro with the increased gel content and degree of methacrylation of the respective macromonomers. In addition, good cell viability and proliferation with a human endothelial cell line (EA.hy926) was observed on GuG-GMA hydrogels [224]. 

The incorporation of galactomannan gum (GaTG) into gelatin-based films showed improved mechanical performance and non-toxicity effect to rMSCs cells and these characteristics confirm their application in wound healing and tissue engineering [225]. In another study, the incorporation of carbon nanotubes (CNTs) in gelatin-TaG (gelatin-TaG) hydrogels showed improved mechanical properties and observed a prevalent non-Fickian diffusion of the drug as well as good cytocompatibility with human keratinocytes [226]. Further, pluripotent embryonic stem cells (ESCs) show self-renewable ability and differentiation into cellular type depending on specific cues (pluripotency). In this case, the locust beam gum (LBG) as a coating material for embryonic stem cells (ESCs) culture has been developed, where undifferentiated mouse ESCs were cultured on commercial LBG to measure its ability for maintaining pluripotent ESCs. The results showed the continuous dome shape with a bright border morphology of ESCs colonies similar to the colonies in co-cultures with pluripotent ESCs and mitotically-inactivated mouse embryonic fibroblast (MEFsi) cells. Further, in short-term cultures, the LBG coating showed ESCs proliferation similar to ESCs cultured in gelatin, while maintaining cell viability. In addition, the mouse ESCs cultured in LBG preserve their tri-lineage differentiation ability and LBG coating promotes long-duration cell growth in an undifferentiated state. Therefore, LBG demonstrates a promising and viable alternative (non-animal) to gelatin-based ESCs culture system as well as time-consuming and laborious parallel MEFsi culture [227]. For periodontal tissue diseases, tetracycline hydrochloride (TCH) drug loaded-core-shell nanofibers of GT/polylactic glycolic acid (PLGA) have been fabricated by coaxial electrospinning. With the effect of GT fraction, these drug loaded-core shell nanofibers showed effective drug release in a controlled manner and sustained for 75 days by showing 19% of burst release within an initial 2 h. In this study, GT had a potential impact on prolonged drug release along with mechanical, cytocompatibility, and antibacterial properties [228].

As described above in previous sections, natural gum-based biomaterials widely used in various tissue engineering applications are summarized precisely in Table 2. 

## 7. Conclusions and Future Perspectives

We have reviewed the important properties and their efficacy in various forms in tissue engineering and regenerative medicine. As described in this review, natural gums are highly abundant sustainable resources, less expensive, and possess excellent biocompatibility, biodegradability with low side effects along with ease of chemical modification and water uptake capacity. Further, natural gums have variability and versatility in their properties. In addition, natural gums might potentially provide naturalistic 3D microenvironment in tissue regeneration, where polysaccharide is a structural component. Natural gums provide structural compatibility much similar to the biological molecules in organisms (in vivo), thereby decreasing the possibility of immune response. [2]. However, natural gums are subjected to high microbial contamination followed by decreased viscosity on storage due to high water content or moisture content and environmental effects, low mechanical properties, uncontrolled hydration rate, environment-dependent processing, and potential antigenicity. Therefore, natural gums alone do not provide all desired properties. For the potential applicability of natural gums in tissue engineering applications, natural gums need to be modified chemically or combined with synthetic polymers or organic/inorganic nanomaterials, and biomolecules (e.g., growth factors) under various processing or synthesizing routes for tunable properties [89,229,230,231]. In this case, the formation of natural gums-based hydrogels is potentially effective in providing native 3D microenvironment (native ECM) by mimicking biologically for cell attachment, proliferation, migration, and differentiation followed by new tissue formation compared to other forms or synthetic biomaterials [89]. The design and development of smart and responsive natural gums-based biomaterials is a challenging task for native cell fate under 3D microenvironment for successive tissue formation. Moreover, this review provides a quite promising future of natural gums in tissue engineering and regenerative medicine, and still there is a need of comprehensive understanding of natural gums, cells, and processing methods, and biological environment in extensive research for successful clinical products, especially in developing polymeric-inks and/or bio-inks for additive manufacturing (i.e., 3D printing, 4D printing (3D printing of programmable inks), or 5D printing as a five-axis system for printing complex structures in multiple dimensions [108]) of tissue scaffolds and/or tissue models for futuristic tissue engineering and regenerative medicine. 

## Figures and Tables

**Table 1:** Natural gums and their properties.

Gum Type	Monosaccharid Composition	Main Chain	Molecular Weight (kDa)	References
Larch gum	Arabinose, galactose	Arabinogalactan	100–120	[69]
Guar gum	Mannose, galactose	Galactomannan	220–250	[70,71]
Locust bean gum	Mannose, galactose	Galactomannan	310	[71,72]
Tara gum	Mannose, galactose	Galactomannan	500	[73]
Cashew gum	Mannose, galactose, glucoronic acid	Galactan	180	[73,74]
Fenugreek seed gum	Mannose, galactose	Mannan	30	[75,76]
Tamarind gum	Glucose, galactose, xylose	Glucan	52.4	[77,78]
Flaxseed gum	Glucose, xylose, galactose, rhamnose	Xylan	285	[79]
Quince seed gum	Galactose, arabinose, xylose	Galactan	150	[69,80]
Gum arabic	Galactose, arabinose, rhamnose, glucoronic acid, 4-O-methylglucoronic acid	Galactan	250–600	[70,81]
Gum karaya	D-galactose, L-rhamnose, D-galacturonic acid	Galactan	9500	[72]
Gum tragacanth	D-galactose, L-fucose, D-xylose, L-arabinose, L-rhamnose	Galactan	840	[73]
Gum ghatti	L-arabinose, D-galactose, D-mannose, D-xylose, D-glucuronic acid	Galactan	12,000	[73,82]
Corn fiber gum	D-xylose, L-arabinose, galactose, glucose, D-glucuronic acid	Xylan	278–394	[76]
Sesbania gum	Mannose, galactose	Galactomannan	241.5–357	[76,83]
Cassia tora gum	Mannose, galactose	Galactomannan	200–300	[84]
Guar gum	Mannose, galactose	Galactomannan	100–200	[12]
Welan gum	L-mannose, L-rhamnose, D-glucose, D-glucuronic acid		100	[85]
Gellan gum	D-glucoronic acid, D-glucose, L-rhamnose		500–2000	[81,86,87]
Xanthan Gum	D-mannose, D-glucose, Pyruvate	D-glucose	2000 to 20,000	[81,88,89]
Tamarind seed gum	D-xylopyranose, D-galactopyranosyl, D-xylpyranose, glucose	D-glucan	115–2500	[90,91,92]
Bael gum	D-galactose, L-arabinose, L-rhamnose, and D-galacturonic	Xyloglucan		[93]
Carrageenan		D-galactose	100–1000	[81]

**Table 2:** Natural gum-based biomaterials for tissue engineering applications.

Constituting Materials		Engineering of Tissue Type	Cell Type	Remarks	References
Gum Type	Other Materials and Biomolecules				
AcG	Crosslinked polyacrylic acid polymer (carbopol), N-vinylpyrollidone (NVP), Moxifloxacin, Glutaraldehyde (GA)	Skin wound tissue	Inflammatory cells	Non-haemolytic, antioxidant, and mucoadhesive in nature	[187]
AcG	SA, ZnONPs, and Glutaraldehyde (crosslinker)	Skin wound tissue	Peripheral blood mononuclear cells (PBMCs) and Sheep fibroblast cells	Significant reduction in toxicity to cells, while maintaining antibacterial and healing effect. Low doses of ZnONPs are beneficial and may reduce undesirable side effects	[188]
AlG	Commercial cream formulation and/or Oligosaccharide (OAG)	Dermal wound healing	Host epithelial cells (skin keratinocytes and fibroblasts)	OAG alone or supplemented to cream formulation exhibits acceleration of wound healing, by promoting neo-blood vessels and collagen	[189]
ArG	HAp urea-formaldehyde (crosslinker)	Bone tissue	C2C12 cells	Scaffolds with 40%–50% of HAp showed highest mechanical properties and supported enhanced biomineralization	[156]
ArG	CS, gelatin, PVA, glutaraldehyde (crosslinker)	Skin tissue	KP-hMSCs	Enhanced mechanical properties and cytocompatibility	[184]
ArG	PCL and Zein	Skin tissue	L929 fibroblast cells	Enhanced mechanical and good antibacterial properties with favorable cell viability	[185]
ArG	PCL, Zein, *C. officinalis*	Skin tissue	L929 fibroblast cells	Desirable mechanical properties, gradual and controlled release of C. officinalis, and better antibacterial and cell viability than PCL/Zein/ArG scaffolds	[186]
ArG	Alg and Recombinant human MG53 protein (rhMG53)	Dermal wound healing		Provided micro-/nanoscale structure, adhesion characteristics, and tunable properties for quick and sustained delivery of rhMG53	[190]
CG	PVA and trypsin	Wound healing	Human PDL fibroblast cell	No cytotoxicity was observed for cells and became bioactive by the immobilization of trypsin	[191]
CGG	Whitlockite (Ca_18_Mg_2_(HPO_4_)_2_(PO_4_)_12_) NPs and dimethyloxallylglycine (an angiogenic drug)	Bone tissue	human umbilical vein endothelial cells	Enhanced in vitro osteogenesis and angiogenesis	[63]
GaTG	Gelatin	Wound and tissue engineering	Rat mesenchymal stem cells (rMSCs)	Enhanced mechanical properties and good cell adhesion with no cytotoxicity	[225]
GeG	ALP, PDA	Bone tissue	MC3T3-E1 cells (osteoblastic cells)	Enhanced ALP-mediated enzymatic mineralization of GeG by the PDA functionalization	[158]
GeG	ALP	Bone tissue	MC3T3-E1 cells and RAW 264.7 monocytic cells	Enhanced osteoblast cell adhesion nd proliferation on hydrogels with Mg-loaded mineral (i.e., mineralized in B–E media)	[159]
GeG	CS, PEG, and APN	Wound healing		Enhanced biocompatibility, entrapment and sustained release of drug, moist nature and antioxidant property	[192]
GeG	HAp	Bone tissue	hASCs	Enhanced mechanical properties, sustained degradation, and cell adhesion and proliferation	[157]
GeG	HAp	Osteochondral tissue	Mouse lung L929 fibroblast cells	Provided temporary load while neotissue formation, good in vivo integration with surrounding tissues and supporting formation of cartilage and bone-like tissue	[183]
GeG	SF and MicroRNAs	Articulate cartilage tissue	BMSCs	Effective and suitable for cell growth and nutrients perfusion; BMSCs-loaded hydrogel transfected with miR-30a promote chondrogenesis of BMSCs with up-regulation of cartilage specific gene	[182]
GeGMA	GelMA	Cartilage tissue	NIH3T3 fibroblast cells	High mechanical strength and cytocompatibility	[175]
GeG		Cartilage tissue	Human nasal chondrocyte cells	High cell entrapment with homogenous distribution, good viscoelastic properties and cytocompatibility	[176]
Oxidized-GeG	CMCS	Cartilage tissue	Chondrocyte cells	Enhanced gelation temperature, mechanical properties, and cell viability	[178]
iGeG-MA	FF-Gen_3_K(WHLPFKC)_16_			Enhanced anti-angiogenesis potential in vitro and in vivo	[179]
GeG-MA	PEG-DMA, sulindac, and vitamin B12	Cartilage tissue	Human fibroblast cells (WI-38 cells)	Better mechanical properties and in vivo cytocompatibility, tunable release of small molecule, whereas no significant difference with large molecules	[180]
GeG		Musculoskeletal tissues/fibrocartilage tissue		Low acyl-GeG (2% *w*/*v*) was found most suitable for cell encapsulation with appropriate mechanics, gelling temperature, and degradation properties	[181]
GeG	GO			Good fracture strength and strain, tensile modulus, and biocompatibility	[214]
GeG		Wound dressing and cartilage tissue		Scaffolds with high surface area to mass ratio and high degradation, improvement in mechanical properties after degradation in SBF	[217]
GeG	PVA	Not specified	Embryonic stem cells (ESCs)	Good stability in aqueous medium and good cell attachment and growth	[218]
GeG	GelMA, PCL, alginate	Not specified	BMSCs	Highly complex structures were achieved; fabrication and sacrificing process did not affect cell viability	[219]
GeGMA	GelMA		NIH3T3 fibroblast cells	3D constructs with tunable microporosity capable of directing cellular responses at millimeter scale (e.g., anisotropic outgrowth)	[213]
GeGMA	Collagen	Vasculogenic differentiation	Bone marrow-derived mesenchymal stem cells (BMSCs)	Effectively promoted BMSCs to differentiate into endothelial cells	[220]
GeGMA		Tissue engineering (not specified)	NIH-3T3 fibroblast cells	Highly tunable degradation and mechanical properties as well as high cell viability	[185]
GeG	Peptides	Soft tissue engineering (not specified)	Human adipose stem cells (hASCs), dermal microvascular endothelial cells (hDMECs) and keratinocytes (hKC) from human adult skin and human osteoblast-like cells SaOs-2	Enhanced mechanical properties and flexibility, cell-adhesiveness of spongy-like hydrogels due to pre-incubation with cell-adhesive protein	[215]
GeGMA	PBS	Intervertebral discs (IVDs) regeneration	Rat lung fibroblast L929 cells	Enhanced mechanical, degradation, and water uptake properties with good cytocompatibility	[162]
GeG	BG	Bone tissue	Rat mesenchymal stem cells (rMSCs)	The incorporation of BG promoted mineralizability and antibacterial properties and differentiation of rMSCs depending on BG-type	[160]
GeG	BG	Bone tissue	Human adipose-derived stem cells (ADMSCs)	Good apatite-forming ability, improved mechanical properties, and cell viability	[161]
GeG	Glycerol and HNTs	Dermal tissue (soft tissue)	Human dermal fibroblast (NHDF-Neo) cells	Tuneable mechanical properties (compressive modulus: 20–75 kPa) and high metabolic activities of cells on 25% HNT loaded-GeG/Gly hydrogels	[120]
GeG	HA and cellular mediators (adipose tissue cells)	Skin tissue	Human microvascular endothelial cells (hAMECs)	Fast wound closure and re-epithelialization, a distinct dermal matrix remodeling, and improved neovascularization was observed	[194]
GeG	HA, Ca^2+^	Wound tissue	Epidermal and dermal cellular fractions (Keratinocytes, fibroblasts, endothelial cells)	Accelerated rate of wound closure and re-epithelialization, including tissue neovascularization	[193]
GeG	EDC	Wound healing	Fibroblast (L929) cells	High reduction in wound size (%) and collagen content	[195]
GeG		Neural tissue	Primary cortical neural cells	Successful printing of complex, layered, and viable 3D cell structures (i.e., brain-like structures)	[211]
GeG	Bioamines (SPD, SPM) and peptide (RGD)	Neural tissue engineering	Human pluripotent stem cell-derived neuronal cells (hPSCs)	Properties mimicking naïve rabbit brain tissue under relevant physiological stress and strain; cell type-specific behavior after functionalization with laminin	[210]
GuG	GMA	Common tissue	Human endothelial cell line (EA.hy926)	Excellent endothelial cell viability	[224]
GeG	PEG		ARPE-19 cell	Promotion of retinal regeneration compared to only GeG and 3 wt.% PEG-GeG could be applied as an alternative for retinal regeneration	[205]
PHGuG		Wound tissue	Young adult mouse colonic (YAMC) epithelial cells	Promotion of colonic epithelial cell wound healing through RhoA activation that occurs downstream of ERK1/2 activation	[196]
GuG	SPI	Bone		Significant improvement of bond strength of SPI adhesives onto porcine bones	[163]
CMGuG	Ethylenediamine, fish collagen, and Ceftazidime drug	Wound healing	NIH3T3 fibroblast cells	Enhanced biocompatibility and antibacterial properties; release of 90–95% Ceftazidime from film after 96 h of incubation at physiological pH	[197]
TaG	Gelatin, CNTs, and salicylic acid	Wound, tissue, and drug delivery	Human keratinocyte (HaCaT) cells	Enhanced mechanical stability, diffusion-mediated drug release, and cytocompatibility	[226]
GT		Bone tissue	Adipose-derived mesenchymal stem cells (ADMSCs)	Supporting and the acceleration of adhesion, proliferation, and osteogenic differentiation of stem cells	[164]
GT	PVA, glutaraldehyde (crosslinker)	Wound healing	human fibroblast AGO cells	Good antibacterial properties against Gram-negative bacteria and cell adhesion and proliferation	[137]
GT	PCL, Cur	Wound healing	Mesenchyme stem cells (MSCs)	Enhanced mechanical properties, sustained release of Cur up to 20 days, and cell adhesion and proliferation for PCL-GT-Cur3%; and significantly fast wound closure with well-formed granulation tissue	[138,139]
GT				Good antibacterial and mechanical properties with suitable biocompatibility and hydrophilic nature	[140]
GT	PVA, SA, and Moxifloxacin drug	Wound dressing		Good biocompatibility with impermeability to microbes and the release of drug via non-Fickian mechanism; best fitting in the Hixson–Crowell model	[198]
GT	*Aloe vera* extract, Al^3+^ as crosslinker	Wound healing	Human fibroblast cells	Excellent wound healing behavior with significant migration rate of fibroblast cells	[199]
GT	Acrylamide, *Terminalia chebula* (TC), AgNPs	Wound healing		Good antibacterial properties against both *B. subtilis* and *E. coli* bacteria	[200]
GT	PLLA	Nerve tissue engineering	Nerve cell (PC12)	Enhanced mechanical properties, cell viability, neurite outgrowth and better cellular phenotype	[212]
XG		Osteoarthritis	ADMSCs	ADMSCs with XG reduced pain associated with osteoarthritis	[168]
XG		Articular cartilage	Chondrocyte cells	XG significantly reversed SNP-reduced cell proliferation and prevented cell early apoptosis rate in a dose-dependent manner	[170]
XG	HAp	Bone tissue		Change in microstructure of gel by mineralization process and enhanced mechanical properties	[44]
XG	BG, CNCs, Borax	Bone tissue	MC3T3-E1 osteoblast cells	Enhanced mechanical properties and cytocompatibility	[165]
XG	SA, HNTs, and CNCs	Bone tissue	MC3T3-E1 osteoblast cells	Enhanced rheological and mechanical properties as well as cytocompatibility	[166]
MWXG		Articular cartilage		Prepared injection of high transparency with low protein and free of endotoxin; significantly protects joint cartilage	[171]
LWXG		Articular cartilage	Rabbit articular chondrocytes	Promoted cell proliferation as well as decreased chondrocyte apoptosis through down-regulation of the protein levels of caspases-3 and bax, and up-regulation of the protein level of bcl-2 in cartilage (in vitro and in vivo)	[173]
XG	GeG/HA	Skeletal muscle tissue (tendon)		Decreased tendon adhesion without reducing tendon strength, rapid swelling, slow degradation, and rapid and close blanketing onto tendon tissue	[174]
XG	CS and Chlorhexidine (CHX)	Wound healing	Human dermal fibroblast cells	Good viscoplastic behavior, cytocompatibility, non-Fickian diffusion mechanism of CHX release in vitro and selective antibacterial behavior against P. gingivalis	[203]
XG	CS and HNTs	Not specified	MC3T3-E1 osteoblast cells	Excellent mechanical properties with good cell viability (in vitro)	[221]
XG	CS, Fe_3_O_4_ MNPs, GDL	Multiple tissues	NIH3T3 fibroblast cell	Enhanced rheological and mechanical properties as well as cytocompatibility	[222]
LBG		Tissue engineering (not specified)	Mouse embryonic stem cell (ESCs)	Coating of LBG promoted mouse ESCs growth in an undifferentiated state	[227]
BFG	HAp	Bone tissue	Osteoblast MG-63 cells	Enhancement in mechanical properties, protein adsorption, antibacterial behavior, cell viability and osteogenic differentiation	[167]
